# Photocatalytic Degradation of Lincosamides in the Presence of Commercial Pigments: Kinetics, Intermediates, and Predicted Ecotoxicity

**DOI:** 10.3390/ijms252413370

**Published:** 2024-12-13

**Authors:** Ewa Masternak, Wojciech Baran, Ewa Adamek

**Affiliations:** Department of General and Analytical Chemistry, Faculty of Pharmaceutical Sciences in Sosnowiec, Medical University of Silesia in Katowice, Jagiellońska 4, 41-200 Sosnowiec, Polandeadamek@sum.edu.pl (E.A.)

**Keywords:** lincomycin, photocatalysis, commercial pigments, ecotoxicity, degradation pathway, ZnO, TiO_2_

## Abstract

Lincomycin belongs to the antibiotics commonly used in veterinary medicine. Its residues are easily spread in the environment because of its physicochemical properties, including resistance to biodegradation and good solubility in water. One of the effective methods for the removal of lincomycin from wastewater is the photocatalytic process, but it is not widely used due to the price of photocatalysts. The aim of this work was to compare the photocatalytic efficiency and the mechanism of lincomycin degradation initiated by UVa radiation in the presence of TiO_2_-P25 and ZnO, as well as in the presence of industrial pigments commonly used in construction and containing TiO_2_. Lincomycin was found to undergo efficient photocatalytic degradation in the presence of a commercial TiO_2_-P25 photocatalyst, industrial pigments containing only anatase, and in the presence of ZnO. On the contrary, industrial pigments containing only rutile or a mixture of rutile and anatase practically did not show any photocatalytic activity. The composition of the solutions after the degradation of lincomycin in the presence of TiO_2_-P25 and ZnO differed significantly. Most of the identified organic degradation products contained conserved pharmacophores, and some of them could have been highly ecotoxic.

## 1. Introduction

Lincosamides are a group of antibiotics commonly used in veterinary medicine. Despite the current trend to reduce the consumption of antibiotics in industrial animal husbandry, their use has remained stable over the second decade of the 21st century [1]. Therefore, particularly large amounts of lincosamides have been found in manure from animals. In the solid fraction of manure, the presence of Lincomycin (LIN) was confirmed at a concentration of almost 10 mg kg^−1^ [2], while in the swine slurry at a concentration exceeding 20 mg L^−1^ [3]. These concentrations cause significant changes in the composition of exposed microbiocoenoses and even significantly exceed the microbial inhibitory concentration (MIC) of pathogens [4]. The good solubility of lincosamides in water and their resistance to biodegradation make them easily spread in the environment. Their residues occur, among others, in soils, surface runoff from agricultural fields, and surface water [5,6,7]. These antibiotics also penetrate groundwater and even deep water [7,8]. Due to their long half-life, they can significantly increase the risk of emergence of antimicrobial resistance in the environment. Lincosamides are used in medicine to combat severe infections, including sepsis caused by streptococci, *Clostridium perfringens*, or *Staphylococcus aureus* [9]. Therefore, the spread of resistance to these antibiotics may also be particularly dangerous to humans [10,11].

Persistent biologically active anthropogenic pollutants (e.g., pharmaceuticals and personal care products, pesticides, azodyes) can be effectively removed from the environmental matrices by degradative methods. These include biological methods [12], advanced oxidation processes [13], or chemical degradation using different reducing agents [14]. One of the effective methods for the removal of lincosamides from wastewater is the photocatalytic process [15,16,17,18,19,20,21,22,23]. The high efficiency of this method has been confirmed in many model experiments using, e.g., TiO_2_, modified TiO_2,_ or modified graphene as photocatalysts [17,19,23]. The most important advantages of the photocatalytic process include the degradation of antibiotics and the deactivation of their antibacterial properties. Moreover, complete mineralization of organic pollutants is also possible. The photocatalytic process allows the use of solar energy, and the catalyst immobilization significantly simplifies the technology and does not generate environmentally harmful waste.

Unfortunately, the commercially used catalyst TiO_2_-P25 (Aeroxide) is practically not active in visible light [24]. The new photocatalysts described in the literature do not have this disadvantage [25,26,27,28,29,30]. However, the economic barrier will most likely prevent their widespread use. In the vast majority of cases, these are substances whose production process is complex and requires the use of expensive substrates, including noble metals and rare earth elements [26,27]. On the other hand, the intensity of UVa radiation in natural sunlight is high enough to consider the practical use of photocatalysts that show activity only in this radiation range. This may be economically more justified than the use of expensive catalysts that are also active in the visible spectrum but require complex preparation.

In our opinion, an alternative may be the use of cheap industrial pigments containing TiO_2_ or ZnO as photocatalysts. We assume that these substances can have enough high photocatalytic activity to be appropriate for their use in the removal of lincosamides from the aquatic environment. The aim of the work was to compare the photocatalytic efficiency and the mechanism of lincomycin (LIN) degradation initiated by UVa radiation in the presence of TiO_2_-P25, ZnO, and industrial pigments commonly used in construction and containing TiO_2_.

## 2. Results and Discussion

### 2.1. Comparison of Catalyst Activity

The pH value of wastewater depends on its origin and, in most cases, is between 4 and 9. Therefore, LIN solutions at pH 4, 7, and 9 were used in the experiments.

Preliminary studies have confirmed that aqueous LIN solutions practically did not undergo UVa-initiated photolysis under experimental conditions. The half-life of LIN degradation due to photolysis ranges from 24 to over 200 days [7].

**Figure 1:**
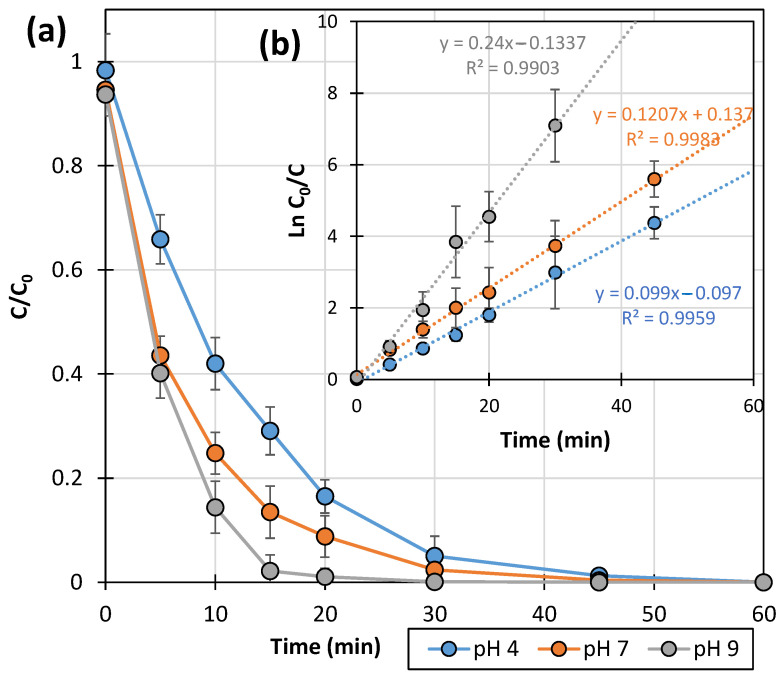
Kinetics of the photocatalytic degradation of LIN (0.1 mmol L^−1^) initiated by UVa irradiation in the presence of TiO_2_-P25 (0.5 g L^−1^) expressed as C/C_0_ = f(t) (**a**) and ln C_0_/C = f(t) (**b**).

Figure 1a shows the change in LIN concentration in the presence of 0.5 g L^−1^ of commercial TiO_2_-P25 photocatalyst during UVa irradiation. For readability, the figure does not include standard deviations.

LIN was found to be degraded during UVa irradiation in the presence of TiO_2_-P25 throughout the pH range studied. The rate of the process increased with increasing pH. The explanation for this phenomenon is related to the structure of the LIN molecule and the physicochemical properties of the mentioned photocatalyst. The pKa value of the LIN solution is approximately 7.5–7.8 [31]. Therefore, in acidic and neutral environments, the nitrogen atoms of the LIN molecule can attach protons, forming cations. In turn, the pH value of the isoelectric point of the TiO_2_-P25 suspension is approximately 6.25. This indicates that in an acidic environment, both catalyst particles and LIN molecules have a positive charge, which does not favor LIN adsorption onto the TiO_2_-P25 surface. In alkaline solutions, the surface charge of TiO_2_-P25 changes, and at the same time, LIN occurs as neutral molecules. Under these conditions, the negative effect of electrostatic repulsion disappears, and the access of LIN to the TiO_2_-P25 surface increases. Moreover, with an increase in pH value, the supply of HO• radicals also increases [32,33].

The degradation dynamics of LIN was also expressed as a function (Figure 1b):

lnC_0_/C = f(t) (1)

In each experiment with TiO_2_-P25, a linear relationship with a high value of the correlation coefficient (R^2^ > 0.99) was obtained. This indicates that the photocatalytic degradation of LIN in the studied pH range follows pseudo-first-order kinetics.

**Figure 2:**
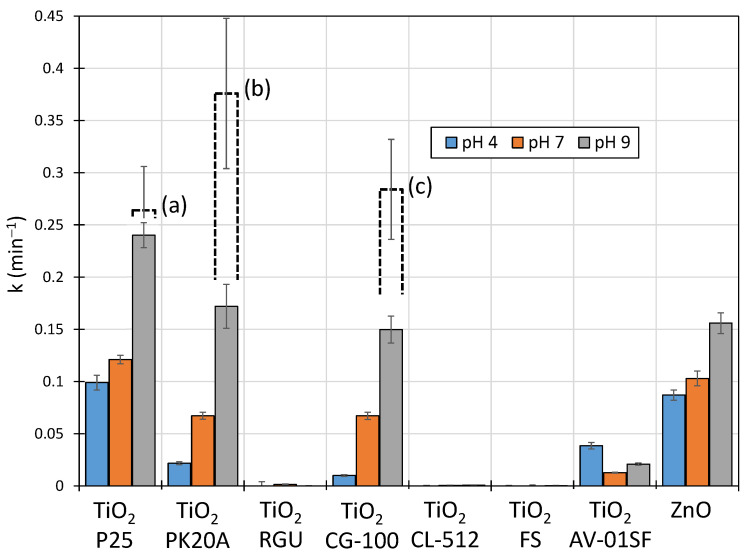
Comparison of the rate constants (k) for the photocatalytic degradation of LIN in the presence of TiO_2_-P25 and industrial pigments with a dose of 0.5 g L^−1^. The dashed line indicates the maximum k-values obtained in the experiment at the catalyst dose of 1.0 g L^−1^ TiO_2_-P25 (**a**), 1.5 g L^−1^ TiO_2_ -PK20A (**b**), and 2.0 g L^−1^ TiO_2_-CG-100 (**c**), respectively.

**Figure 3:**
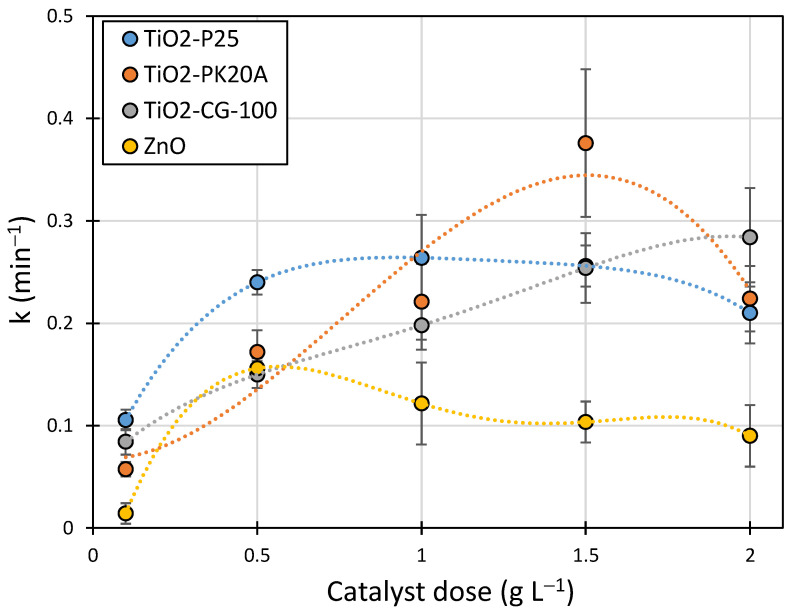
Effect of the catalyst dose on the degradation rate of LIN.

Figure 2 shows the rate constant (k) values for the photocatalytic degradation of LIN carried out in the presence of industrial pigments used in construction and, for comparison, in the presence of TiO_2_-P25. Detailed characteristics of the pigments used are given in Table 1. The dashed lines refer to the maximum k-values obtained for LIN solutions of pH 9 with catalyst doses in the range of 0.1–2.0 g L^−1^ (Figure 3).

In an acidic (at pH 4) and neutral environment, the highest k-value was obtained for the reaction carried out in the presence of TiO_2_-P25. Under these conditions, the degradation of LIN in the presence of ZnO occurred at a slightly slower rate. Industrial pigments containing TiO_2_-anatase were characterized by significantly lower activity than TiO_2_-P25. In turn, pigments containing TiO_2_-rutile showed practically no photocatalytic activity in any pH range tested.

In an alkaline environment (at pH 9) with a catalyst dose of 0.5 mg L^−1^, TiO_2_-P25 also had the highest photoactivity. ZnO and TiO_2_-anatase with the trade names PK20A and CG-100 had approximately 30% lower photoactivity. However, in the case of ZnO, a significant problem was the homogenization of the solution with the catalyst. Because of this, these solutions required much more intensive mixing. In addition, ZnO in alkaline solutions can undergo photochemical corrosion [34], and zinc compounds exhibit ecotoxic effects [35,36,37]. These aspects constitute significant limitations for the application of ZnO in the removal of anthropogenic pollutants.

Increasing the dose of these catalysts that showed high photoactivity resulted in an additional increase in the k-value (Figure 2 and Figure 3—dashed line). This change was particularly significant in solutions with pH 9, that is, in an environment with a pH similar to that of natural sewage generated during animal breeding. In the case of TiO_2_-P25, the rate of LIN degradation increased by approximately 10%, while in the presence of pigments with trade names PK20A and CG10, it increased by approximately 120 and 90%, respectively, compared with those experiments in which a dose of 0.5 g L^−1^ was used at the same pH. This indicates that the use of higher doses of these industrial pigments significantly increased the efficiency of the photocatalytic degradation of LIN. Due to their low price, this fact may be of great practical importance. In the case of ZnO, increasing the dose from 0.5 to 2 g L^−1^ did not increase the rate of LIN photodegradation (Figure 3).

### 2.2. Identification of LIN Degradation Products

**Figure 4:**
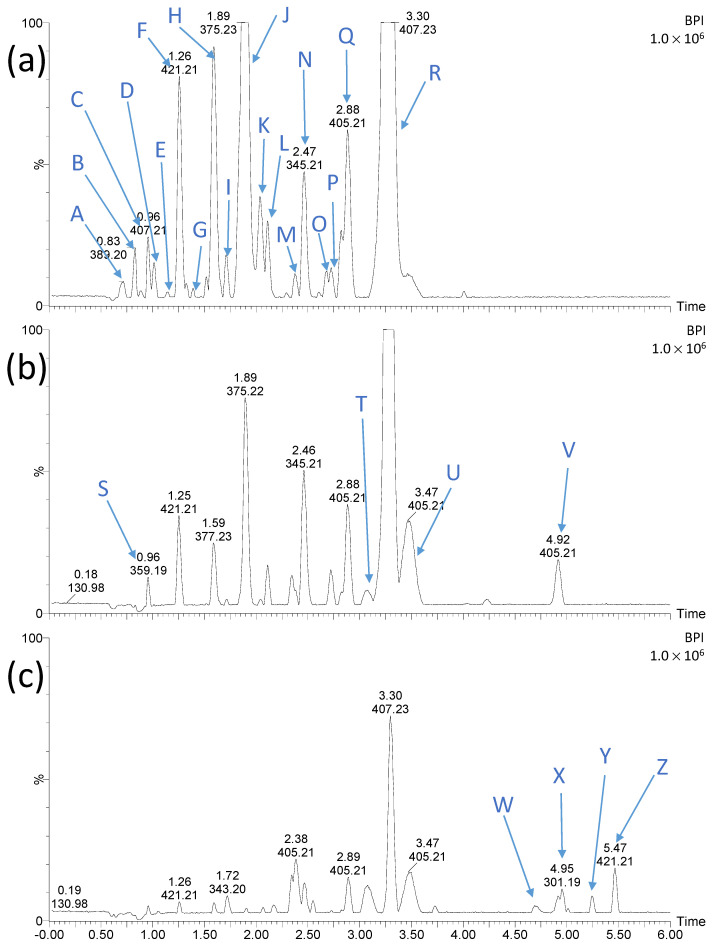
Chromatograms of LIN solutions (0.1 mmol L^−1^) after UVa irradiation in the presence of TiO_2_-P25 for 15 min at pH 4 (**a**), 7 (**b**), and 9 (**c**). The letters symbols are explained in Figure 5.

Chromatograms of LIN solutions irradiated for 15 min at different pH in the presence of TiO_2_-P25 are shown in Figure 4.

It was observed that the changes in pH have a significant effect on the retention time (t_R_) and the intensity of the recorded peaks. This is closely related to differences in the degree of LIN degradation. In an alkaline environment, this process occurs more than twice as fast as in an acidic and neutral environment (Figure 1 and Figure 2). Furthermore, with the increasing basicity of the irradiated solutions, the number and intensity peaks of organic degradation products (ODPs), which were less polar than LIN, increased (Figure 4c). This may indicate that these ODPs (substances) were formed mainly in an alkaline environment or that their subsequent degradation was slowed down under these conditions. As mentioned in Section 2.1, the reaction environment affects the surface charge of the photocatalyst particles and the degree and type of ionization of the organic compounds present in the solution. The slowdown in the photocatalytic degradation of these ODPs (ions) may be due to their repulsion from the negatively charged catalyst surface.

**Figure 5:**
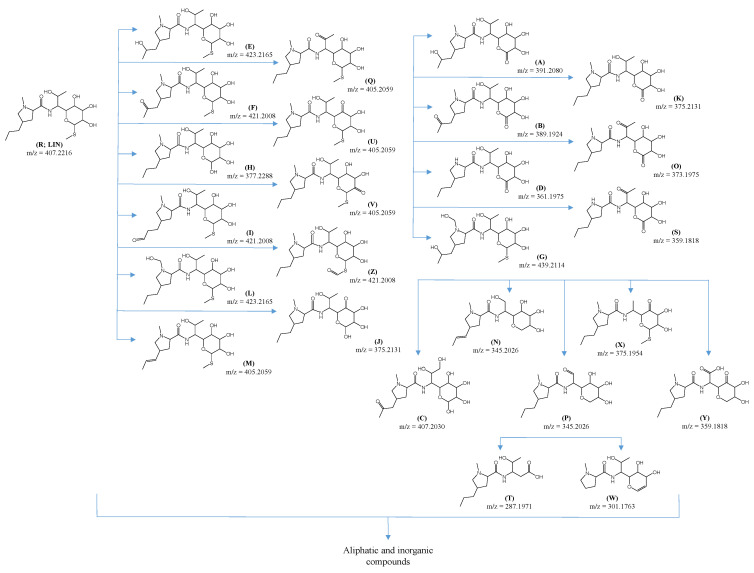
Identified ODPs formed after UVa irradiation of LIN solution (0.1 mmol L^−1^) in the presence of TiO_2_-P25 at pH 4, 7 and 9.

Figure 5 shows probable structural formulas of 25 compounds that correspond to ODPs of LIN recorded as chromatographic peaks. These are substances with a preserved heterocyclic ring and a sugar ring (except for the T product). These ODPs are mainly products of oxidation that result in the introduction of at least one additional hydroxyl group (marked A, C, E, G, and L), substitution or elimination of methylthio group (A, B, C, D, H, J, K, N, O, P, S, W, and Y), and/or oxidation of a hydroxyl group to a carbonyl group (B, C, F, I, O, P, Q, S, U, X, Y, and Z). In turn, those ODPs (marked M, N, and W) containing multiple bonds could have been formed by the elimination of hydrogen or alkyl groups. Some of the proposed ODPs have also been described by other authors as products of various advanced oxidation processes [16].

Due to the analytical procedure used, the potentially formed aliphatic compounds were not identified.

**Figure 6:**
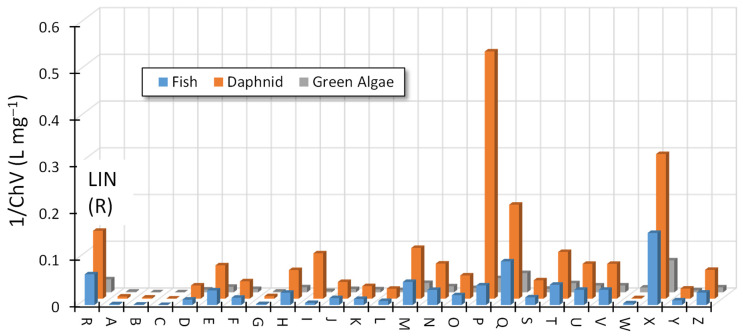
Predicted chronic toxicity (ECOSAR^®^) of identified ODPs of LIN (0.1 mmol L^−1^) toward three groups of organisms. Photocatalyst: TiO_2_-P25.

The identified ODPs of LIN have a preserved pharmacophore of this antibiotic and, therefore, may be biologically active and can negatively affect the aquatic environment. Figure 6 presents the predicted chronic toxicity of these products toward fish, daphnids, and algae.

Most ODPs showed toxicity, although less than the initial antibiotic. Some ODPs, especially those more polar than LIN (marked A, B, C, and G) and less polar (W), should practically not show any toxicity. However, it is possible that products (P, Q, and X) that remained in post-reaction solutions were characterized by ecotoxicity significantly higher than that of LIN. For example, the predicted toxic effect of the substance marked as P toward daphnids occurred already at a concentration of <2 mg L^−1^. Most likely, such a high concentration of this ODP will not be possible during routine photocatalytic wastewater treatment. However, it is suggested to control the actual ecotoxicity of effluent after photocatalytic treatment of wastewater containing lincosamides.

**Figure 7:**
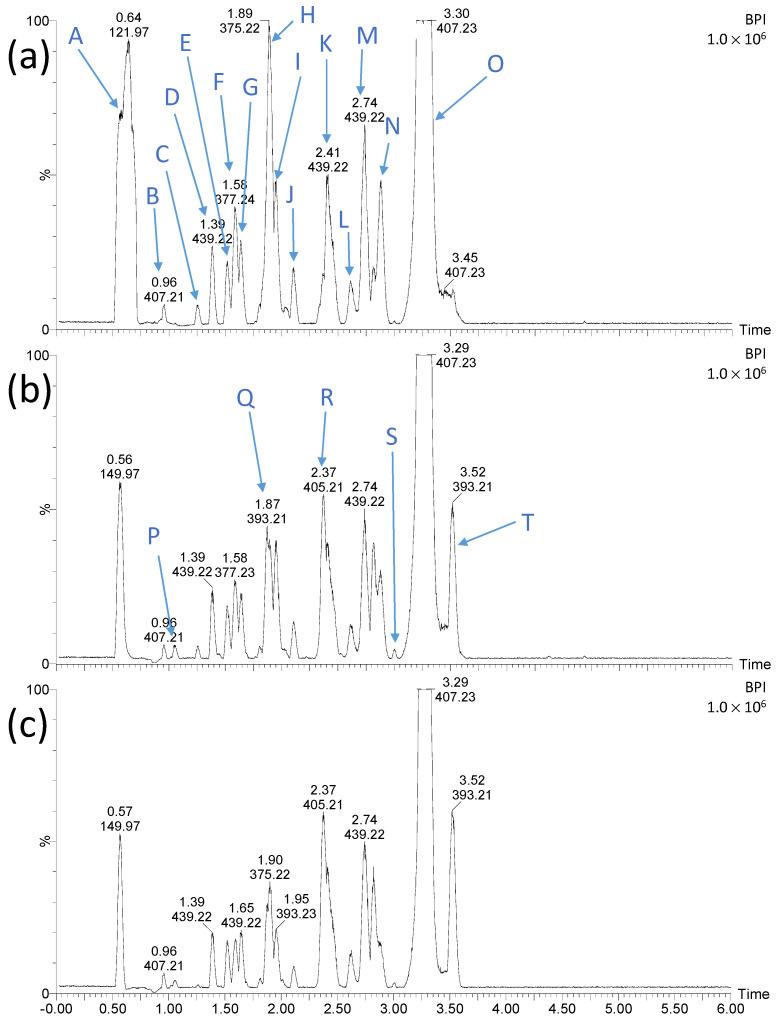
Chromatograms of LIN solutions (0.1 mmol L^−1^) after UVa irradiation in the presence of ZnO for 15 min at pH 4 (**a**), 7 (**b**), and 9 (**c**). The letters symbols are explained in Figure 8.

Chromatographs of LIN solutions irradiated for 15 min at different pH in the presence of ZnO are shown in Figure 7.

For this catalyst, the change in pH of solutions did not have such a significant effect on the t_R_ and the intensity of peaks recorded on chromatograms. Furthermore, no peaks with t_R_ > 3.52 min were recorded, which corresponded to ODPs much less polar than LIN and were recorded during photocatalysis with TiO_2_-P25 (Figure 4). These compounds were also not identified in LIN solutions irradiated for more than 15 min.

**Figure 8:**
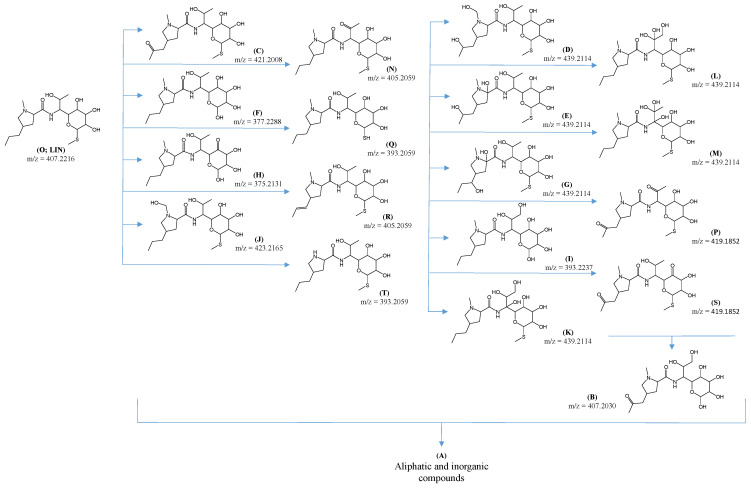
Identified ODPs formed after UVa irradiation of LIN solution (0.1 mmol L^−1^) in the presence of ZnO at pH 4, 7, and 9.

The proposed structural formulas of the identified ODPs of LIN formed after 15 min of UVa irradiation are shown in Figure 8. The mechanism of the photocatalytic reaction carried out in the presence of ZnO assumes the participation of HO• radicals, identically as with TiO_2_-P25. The identified hydroxylation, dehydrogenation, or elimination products probably have been formed as a result of the oxidative action of these radicals. However, as many as 9 out of the 18 identified ODPs (that is, E, G, I, K, L, M, P, S, and T) were not present in solutions after photocatalytic degradation of LIN in the presence of TiO_2_-P25. This may indicate that, under the experimental conditions, the identified ODPs formed in the presence of ZnO underwent subsequent reactions, leading to their further decomposition but more slowly. These ODPs probably had a lower affinity to ZnO than to TiO_2_-P25. It cannot be excluded that this phenomenon was due to the much higher zeta potential of ZnO than TiO_2_-P25 [37]. The occurrence of a steric effect related to the surface structures of both catalysts could also be the reason for the observed effect.

**Figure 9:**
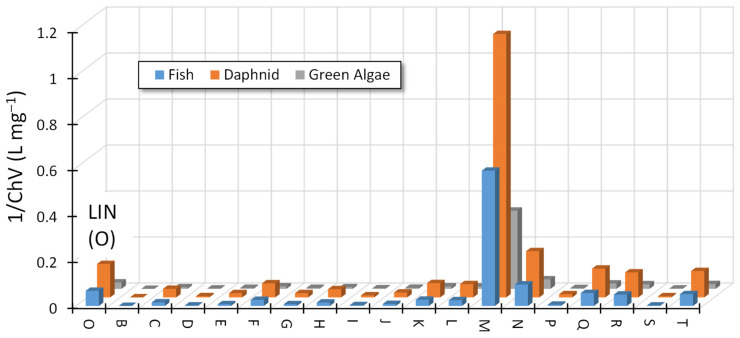
Predicted chronic toxicity (ECOSAR^®^) of identified ODPs of LIN (0.1 mmol L^−1^) toward three groups of organisms. Photocatalyst: ZnO.

The predicted ecotoxicity of ODPs formed during LIN degradation in the presence of ZnO was surprising. For example, the ecotoxicity of the compound marked with the letter “M” was more than five times higher than that of the initial LIN (Figure 9). This product was present in each of the solutions, i.e., at pH 4, 7, and 9. Product N had a slightly higher predicted ecotoxicity compared with that of LIN, and products Q, R, and T were characterized by ecotoxicity similar to that of the initial antibiotic.

Most of the identified ODPs of LIN formed in the presence of TiO-P25 and ZnO had a preserved pharmacophore [9]. Therefore, after introduction into the environment, they can significantly affect microbiocenosis. This unfavorable phenomenon can promote the development of resistance to lincosamides among environmental microorganisms. For this reason, the real effectiveness of using photocatalysis to remove lincosamides should additionally be confirmed by appropriately selected ecotoxicity bioassays and/or software programs, which allow us to estimate ecological toxicity using predictive models [38,39].

**Table 1:** Characteristics of the compounds used as photocatalysts in the experiments.

Trademark	Manufacturer	Chemical Formula (Purity)	Polymorphic Forms	Specific Surface Area (m^2^g^−1^)	Approximate Price (USD kg^−1^) /min. Order (kg)
Aeroxide^®^ P25	Evonic Industries AG, Essen, Germany	TiO_2_ (99.5%) ^1^	Anatase (80%), Rutile (20%) ^1^	35–65 ^1^	2850/0.1 ^2^ 5/1000 ^3^
Pretiox^TM^ PK20A	Precheza a.s, Přerov, Czech Republic	TiO_2_ (>92%) ^4^	Anatase (80%) ^4^	70–110 ^4^	7.72/25 ^5^
Pretiox^TM^ RGU	Precheza a.s, Přerov, Czech Republic	TiO_2_ (>94%) ^4^	Rutile (98%) ^4^	No data	3.72/25 ^5^
Pretiox^TM^ CG100	Precheza a.s, Přerov, Czech Republic	TiO_2_ (>92.5%) ^4^	Anatase ^4^	70–110 ^4^	7.72/25 ^5^
Tiomax^TM^ CL-521	Precheza a.s, Přerov, Czech Republic	TiO_2_ (>95%) ^4^	Rutile ^4^	No data	5.24/25 ^5^
Pretiox^TM^ FS	Precheza a.s, Přerov, Czech Republic	TiO_2_ (>93%) ^4^	Anatase, Rutile (60%) ^4^	No data	3.48/25 ^5^
Pretiox^TM^ AV-01SF	Precheza a.s, Přerov, Czech Republic	TiO_2_ (>98%) ^4^	Anatase ^5^	No data	3.48/25 ^5^
Zinc oxide	Sigma-Aldrich, Saint Louis, MO, USA	ZnO (>99.0%) nr cat: 96479	Hexagonal wurtzite ^6^	3.7 ± 0.1 ^7^	450/0.1 ^2^ 0.79/1000 ^3^

^1^ https://products.evonik.com; ^2.^ www.sigmaaldrich.com; ^3.^ www.alibaba.com; ^4.^ https://www.precheza.cz; ^5.^ http://www.zwukso.pl; ^6^ [40]; ^7^ [41].

## 3. Materials and Methods

### 3.1. Materials

The characteristics of the commercial products used for the photocatalytic degradation of LIN are given in Table 1. TiO_2_-P25 was treated as a reference compound.

### 3.2. Photocatalytic Process

**Figure 10:**
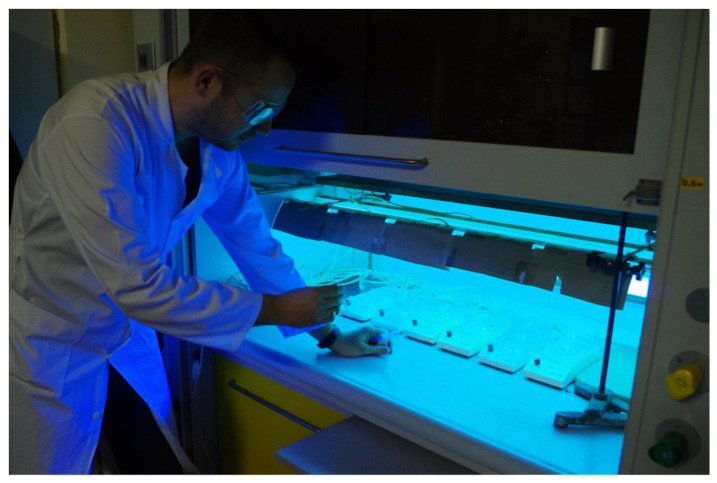
The test stand for irradiation of LIN solutions.

LIN (lincomycin hydrochloride, purity > 95%) was purchased from Sigma Aldrich (Sigma-Aldrich, Saint Louis, MO, USA). The LIN solution (0.1 mmol L^−1^) in deionized water (conductivity < 6µS cm^−1^) was used in all experiments. The antibiotic solution (100 mL) was poured into six glass crystallizers (500 mL, with an inner diameter of 115 mm). Then, in the dark, weighed amounts of catalysts (Table 1) were added to the crystallizers, and the appropriate pH was adjusted using HCl (1.0 mol L^−1^) or NaOH (1.0 mol L^−1^). The pH of the solutions was controlled using a multimeter HQ440d multi (HACH). Solutions with catalysts were mixed for approximately 20 min in the dark and then, without interrupting the mixing, irradiated with UVa radiation with an intensity of 13.6 W m^−2^. The intensity of radiation in the UVa range was controlled using a quantum-photo radiometer DO972 (Delta OHM). At set time intervals, the aliquots were taken from crystallizers and filtered using syringe filters (25 mm nylon welded syringe filter 0.45 µm, Labfil). Throughout the experiment, the irradiated solutions had free contact with atmospheric air, and their temperature was 295 ± 1 K. The test stand used for irradiation of samples is shown in Figure 10.

### 3.3. UPLC Analysis

Analysis was carried out using the UPLC method (ACQUITY UPLC I Class System, Waters, Milford, MA, USA; column: ACQUITY UPLC BEH C18, 130 Å, 1.7 µm, 2.1 mm × 100 mm; detector: QTof Xevo G2-XS Waters, Milford, MA, USA). The details of the chromatographic separation are given in Table 2.

The ODPs of LIN were identified by comparing chromatograms recorded on the QTof detector for samples before and after UVa irradiation. The protonated monoisotopic molecular masses (M + H^+^) and their fragmentation spectra (with fragmentation energies ranging from 10 to 25 eV) were determined for the peaks corresponding to the ODPs of LIN. The structural formulas of the identified ODPs were drawn using the ChemDraw Std (ver. 23.1.1) with Analysis package (iCambridgeSoft). Low-molecular aliphatic reaction products were not identified.

### 3.4. Toxicity Prediction

The potential ecotoxicity of the identified ODPs of LIN was estimated using the ECOSAR (Ecological Structure Activity Relationships) Application 2.2 [42]. ECOSAR can predict short-term and long-term ecotoxic effects using Structure–Activity Relationships (SARs) and Quantitative Structure–Activity Relationships (QSARs). Chronic toxicity was expressed as the chronic toxicity value (ChV) and was defined as the geometric mean of the no observed effect concentration (NOEC) and the lowest observed effect concentration (LOEC). Mathematically, ChV was expressed as 10^([log(LOEC × NOEC)]/2)^. ECOSAR software (ver. 2.2) allows the estimation of the highest chronic toxicity of compounds in relation to aquatic organisms at three trophic levels, i.e., green algae, fish, and daphnids. If the ODPs caused multiple effects, the lowest toxicity concentration was selected as the most conservative estimate based on the precautionary principle.

## 4. Conclusions

LIN can undergo efficient photocatalytic degradation in the presence of the commercial TiO_2_-P25 photocatalyst, industrial pigments containing only anatase, and ZnO. When each of these compounds was used, the degradation rate of LIN increased with increasing pH. Industrial pigments that contained only rutile or a mixture of rutile and anatase practically did not show any photocatalytic activity.

In the post-reaction solutions, mainly LIN oxidation products were detected, containing introduced oxygen atoms, removed hydrogen atoms or alkyl groups, and thioalkyl groups substituted with oxygen. The exposure time and pH had a significant effect on the composition of the post-reaction solutions.

Most of the identified ODPs contain conserved pharmacophores, and some of them may be highly ecotoxic. For this reason, it is suggested to control the actual ecotoxicity of the effluent after photocatalytic treatment of wastewater containing lincosamide antibiotics. Taking into account economic considerations, the use of industrial pigments (especially Pretiox^TM^ CG-100 and Pretiox^TM^ PK20A) for the LIN removal from aqueous solutions can provide satisfactory results at much lower costs than the analogous use of a commercial Aeroxide^®^ TiO_2_-P25. This is a cost-effective solution, although it requires the use of higher doses of these pigments. The use of ZnO as a photocatalyst should be limited because of its potential ecotoxicity.

## Figures and Tables

**Table 2:** Detailed data for the analytical procedure and the mobile phase compositions.

Component A	H_2_O (for LC-MS Chromasolv^®^; Fluka), with 0.01% HCOOH (98-100%, for LC-MS, LiChropur^®^; Sigma-Aldrich)
Component B	CH_3_CN (for LC-MS LiChrosolv^®^; Sigma-Aldrich) with 0.01% HCOOH
Gradient (min; % A)	0.0–95, 5.5–85, 6.0–70, 7.0–95,
Flow rate	0.350 mL min^−1^
Sample volume	1 µL and 5 µL
Column temperature	35 °C
Source	ES+
Scan time	0.1 s
Start mass	50.0 Da
End mass	600.0 Da
Operating mode	ms and ms/ms
Collision energy	0–25 eV
Reference compound	Leucine Enkephalin single point (ms)
Software	MassLynx v4.1

## Data Availability

The data presented in this study are available on request from the corresponding author. The data are not publicly available due to the very large size of the chromatographic files.
